# Supplementary material for: Genome-Wide Analysis and Characterization of the SDR Gene Superfamily in Cinnamomum camphora and Identification of Synthase for Eugenol Biosynthesis
Source: Int J Mol Sci. 2024 Sep 19;25(18):10084. doi: 10.3390/ijms251810084 (PMC11432319; doi:10.3390/ijms251810084)
Supplement: Supplementary file 1 [file ijms-25-10084-s001.zip › Supplementary Figure.pdf]

Article

Genome-Wide Analysis and Characterization of the SDR Gene Superfamily in *Cinnamomum camphora* and Identification of Synthase for Eugenol Biosynthesis

Yueting Zhang[1,2], Chao Fu,[1,2,] Shifang Wen[1], Ting Zhang[1], Xindong Wang[1,2*]

1 Camphor Engineering and Technology Research Center of National Forestry and Grassland Administration, Jiangxi Academe of Forestry, Nanchang 330032, China;

2 Jiangxi Provincial Key Laboratory of Improved Variety Breeding and Efficient Utilization of Native Tree Species (NO.2024SSY04091), Jiangxi Academe of Forestry, Nanchang 330032, Jiangxi, China

* Correspondence: x_wangxindong@jxlky.cn; Tel.: : +86-0791-83987692

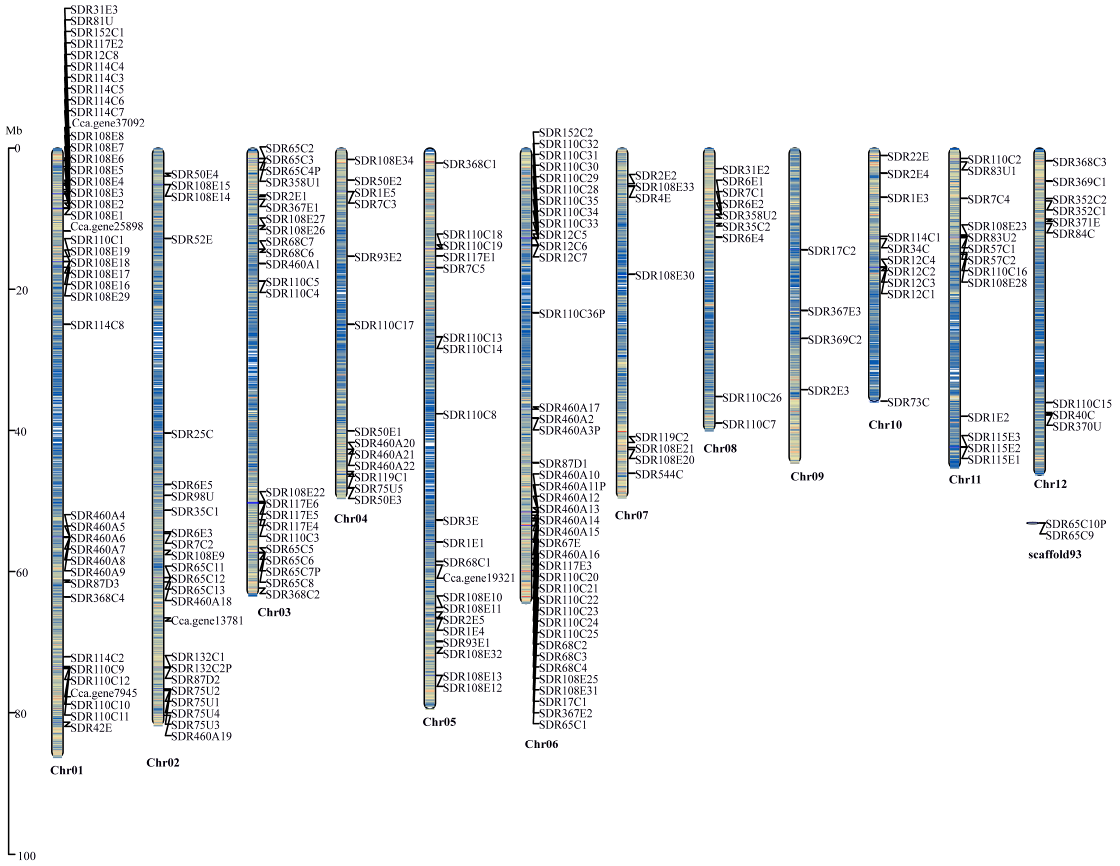

Supplementary Figure S1. Mapping of *CcSDR* genes onto pseudochromosomes of *C. camphora.*

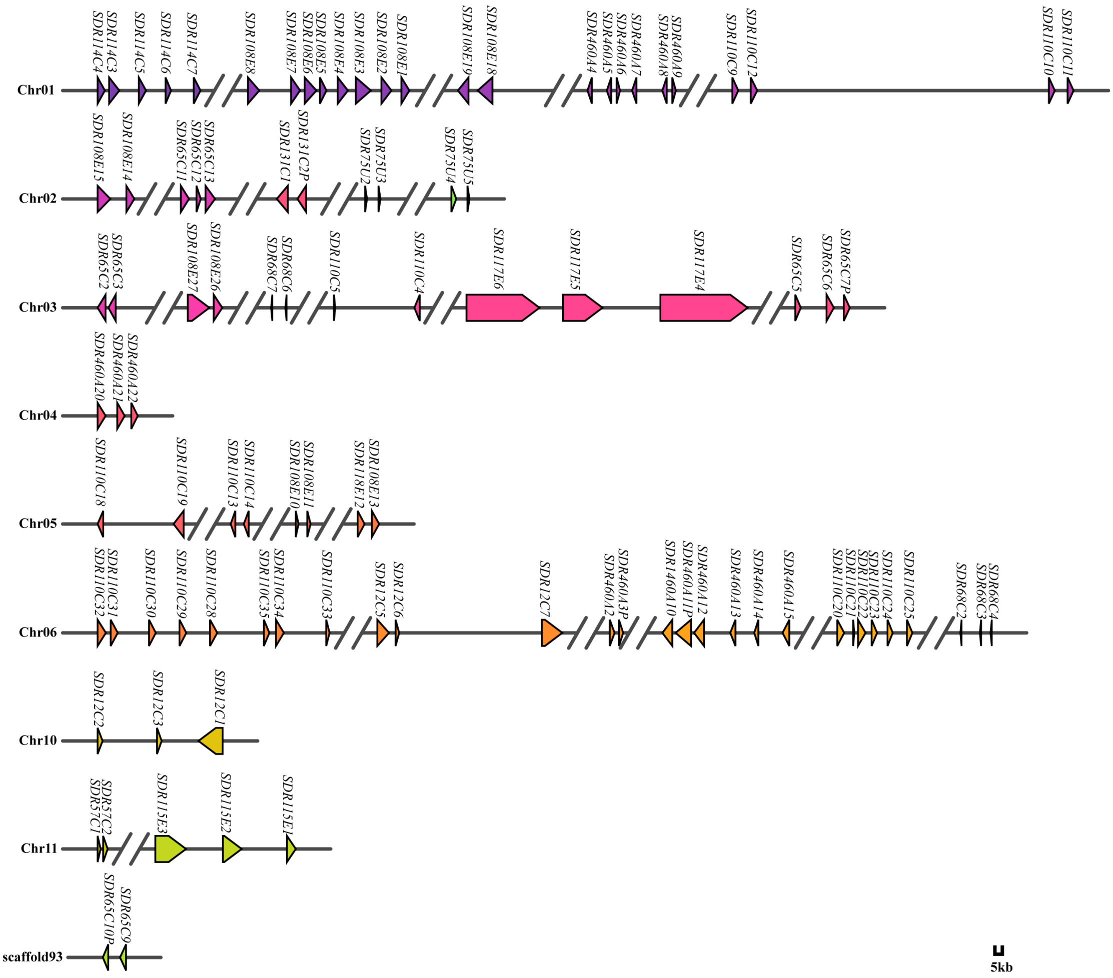

Supplementary Figure S2. Tandem array of some *CcSDR* genes in pseudochromosomes of *C. camphora*.

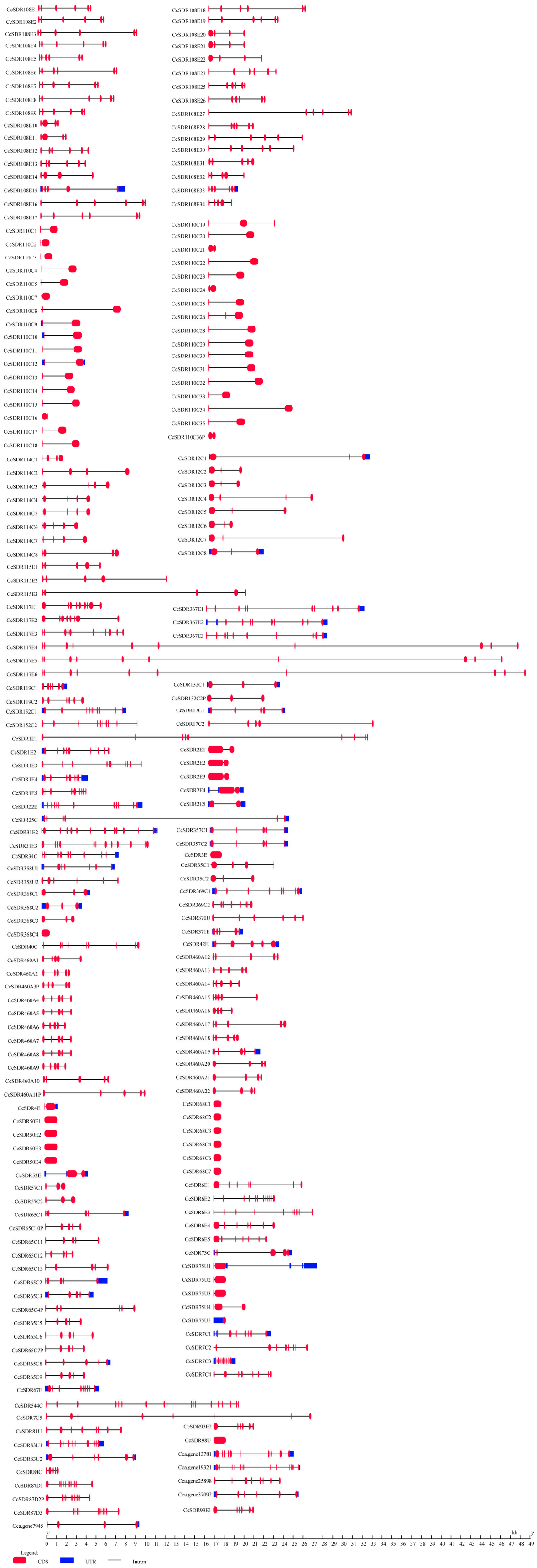

Supplementary Figure S3. Genomic organization of *CcSDR* genes in *C. camphora* genome.

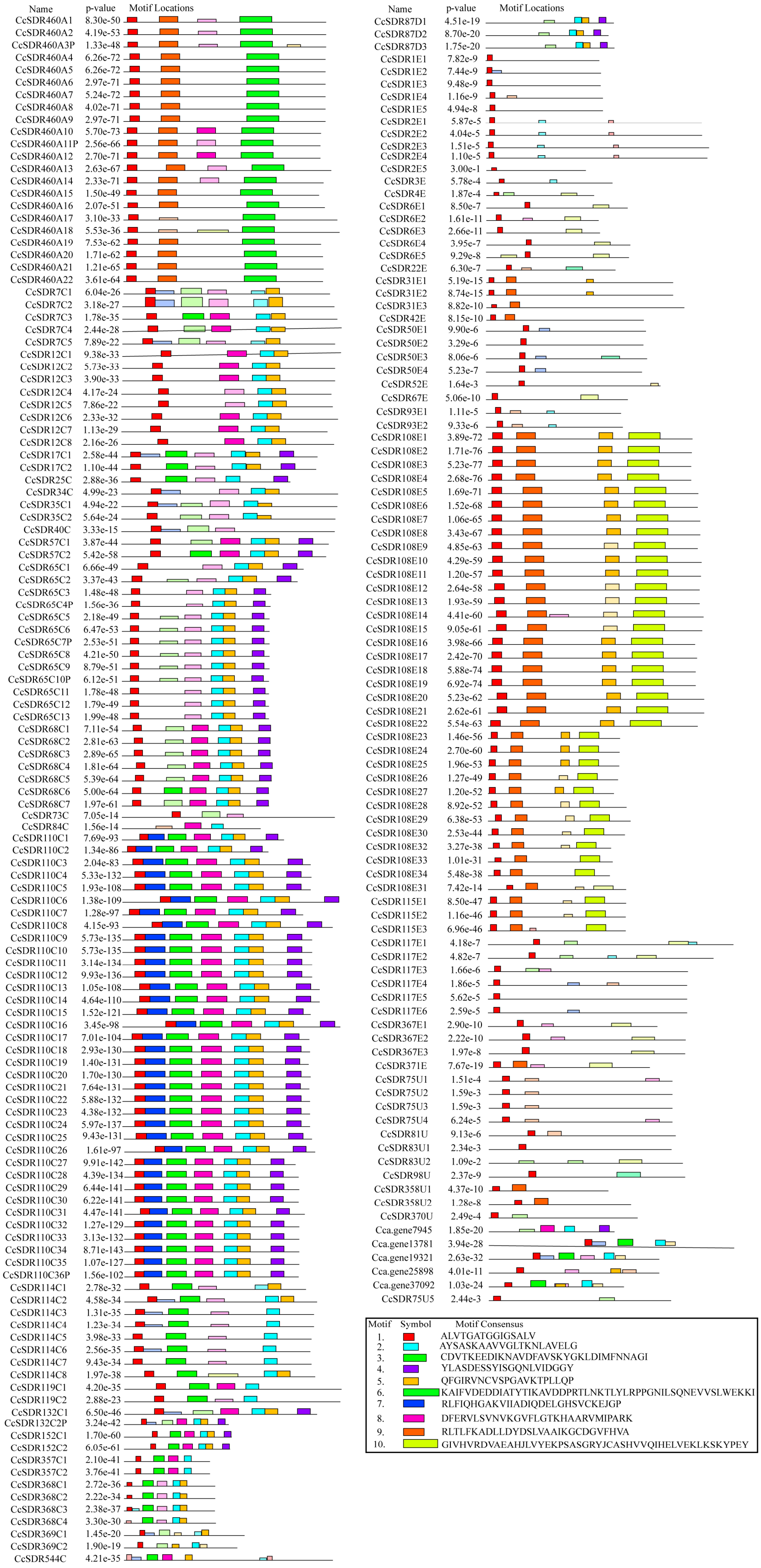

Supplementary Figure S4. Arrangement of ten conservative motifs in the CcSDR superfamily.

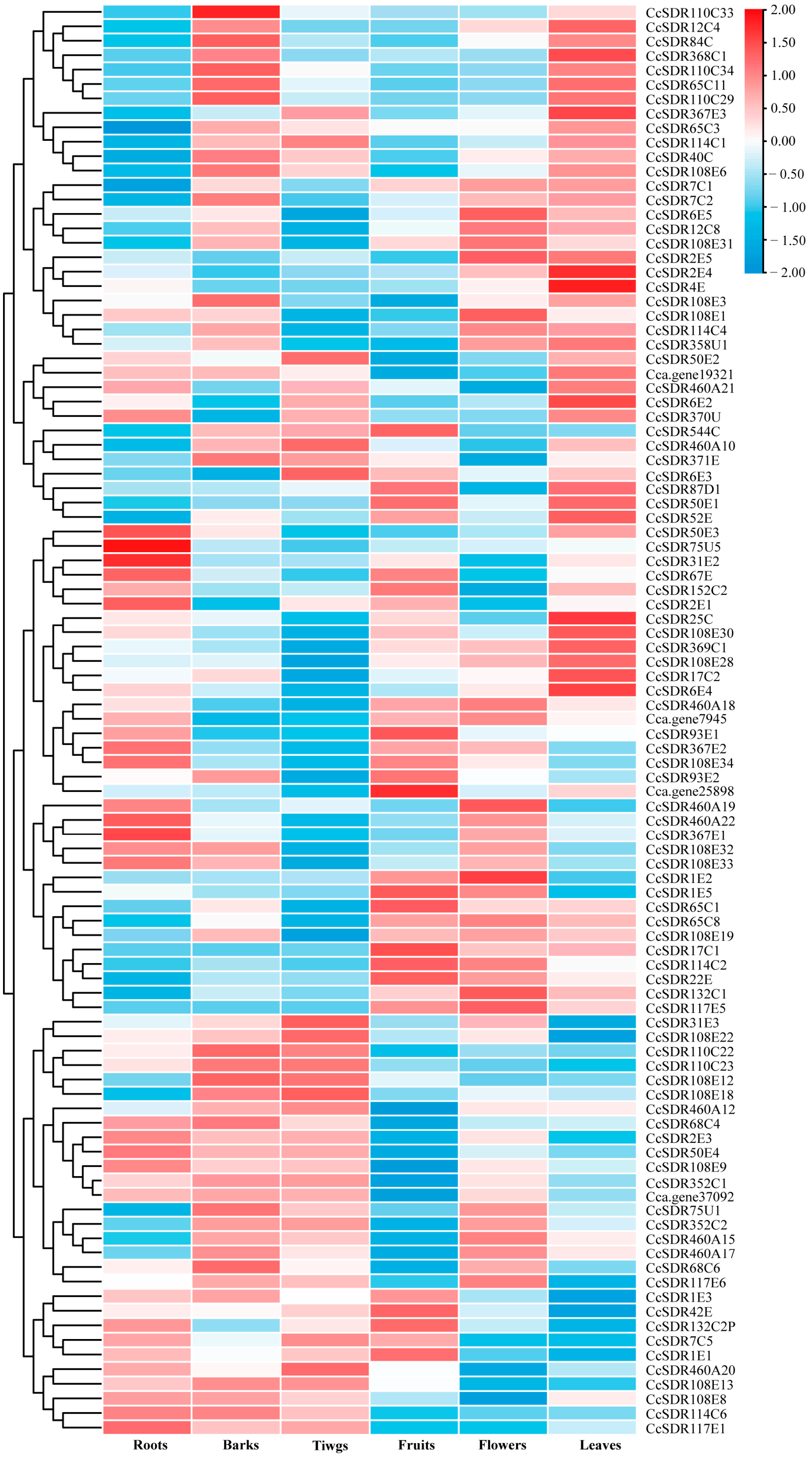

Supplementary Figure S5. Differential expression of *CcSDR* genes across six organs of *C. camphora.* The data of heat map had been manipulated using log2 transformation to enhance the visualization of expression differences and to normalize the data across the different samples.

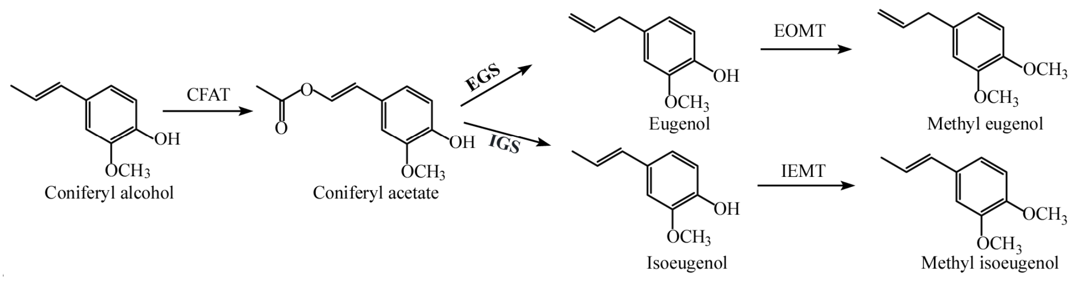

Supplementary Figure S6. The biosynthesis pathway of methyeugenol and methylisoeugenol in plant. CFAT: Coniferyl alcohol acyltransferase; EGS: eugenol synthase; IGS: isoeugenol synthase; EOMT: Eugenol O-methyltransferase; IEMT: (Iso)eugenol O-methyltransferase.

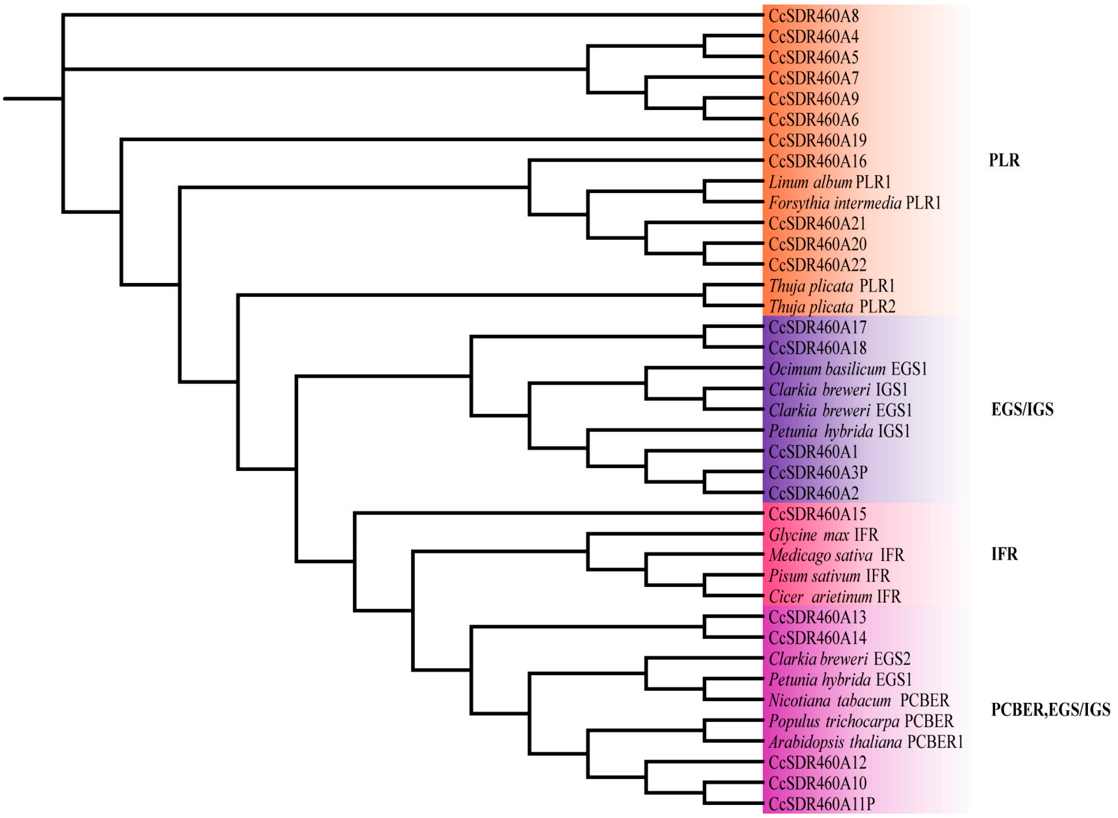

Supplementary Figure S7. Phylogenetic tree analysis of the 460A (PIP) family from *C. camphora* and other plants. *Linum album* PLR1 (UniProtKB: Q4R0I0); *Forsythia intermedia* PLR1 (P93143); *Thuja plicata* PLR1 (Q9LD14); *T. plicata* PLR2 (Q9LD13); *Ocimum basilicum* EGS1 (Q15GI4); *Clarkia breweri* EGS1 (D0VWT0); *C. breweri* EGS2 (B2WSN0); *C. breweri* IGS1 (B2WSM8); *Petunia hybrida* EGS1 (B2WSN1); *P. hybrida* IGS1 (Q15GI3); *Glycine max* IFR (I1LHU6); *Medicago sativa* IFR (P52575); *Pisum sativum* IFR (P52576); *Cicer arietinum* IFR (Q00016); *P. trichocarpa* PCBER (B9HRL7); *A. thaliana* PCBER (Q9T030); Nicotiana tabacum PCBER (B6VRE8).

```
                        *        20         *        40         *        60         *        80
CcSDR460A1 : --MES-QSKMLIFGATGYLGKYMVKASVSMGHPTYVYVRPNTPATNSSRLELLHEFESMGVKILQGDLDEHDKLVDAIKQ :  77
ObEGS1     : MEENGMKSKILIFGGTGYIGNHMVKGSLKLGHPTYVFTRPNSSKTT-----LLDEFQSLGAIIVKGELDEHEKLVELMKK :  75
PhEGS1     : ---MAEKSKILIIGGTGYIGKFVVEASAKAGHPTFVLVR-ESTVSDPAKGKIVESFNNSGVTILYGDLYDHESLVKAIKQ :  76
CbEGS1     : ------MEKIIIYGGTGYIGKFMVRASLSFSHPTFIYARPLTPDSTPSSVQLREEFRSMGVTIIEGEMEEHEKMVSVLKQ :  74
CbEGS2     : ---MGSKSKILIIGGTGYIGKFIVEASVKEGHPTFALVR-ETTVSDPVKGKLVEKFQNLGVSLLYGDLYDHDSLVKAIKQ :  76
CbIGS1     : ------MEKIIIYGGTGYIGKFMVRASLSFSHPTFIYARPLTPDSTPSSVQLREEFRSMGVTIIEGEMEEHEKMVSVLRQ :  74
PhIGS1     : --MTTGKGKILILGATGYLGKYMVKASISLGHPTYAYVMPLKKNSDDSKLQLLKEFESLGVTIFYGELSEHDKLVAVFKE :  78
                   GxxGxxG

                        *       100         *       120         *       140         *       160
CcSDR460A1 : VDVVISTLAVPQHLEQLKIIDAIKDAGNIKRFVPSEYGNEVDRVSGLPPFQA-LLDNKKVIRRATEAAGIPHTYISANSF : 156
ObEGS1     : VDVVISALAFPQILDQFKILEAIKVAGNIKRFLPSDFGVEEDRINALPPFEA-LIERKRMIRRAIEEANIPYTYVSANCF : 154
PhEGS1     : VDVVISTVGQMQLADQTKIIAAIKEAGNIKRFFPSEFGMDVDKVNAVEPAKS-TFAIKVQIRRAIEAEGIPYTYVSSNCF : 155
CbEGS1     : VDIVISALPFPMISSQIHIINAIKAAGNIKRFLPSDFGCEEDRIKPLPPFES-VLEKKRIIRRAIEAAALPYTYVSANCF : 153
CbEGS2     : VDVVISTVGFMQIADQTKIIAAIKEAGNVKRFFPSEFGNDVDHVNAVEPAKSVAFAVKANIRRAVEAEGIPYTYVASNCF : 156
CbIGS1     : VDVVISALSVPMYPSQLLIIDAIKAAGNIKRFLPSEFGSEEDRIKPLPPFES-VLEKKRIIRRAIEAAELPYTYVSANCF : 153
PhIGS1     : VDIVISTLAVPQYLEQLKVIEAIKEAGNIKRFVPSEFGNEVDRVRALPRFQA-VLDNKKKIRRATEAAGIPFTFVSANSL : 157

                        *       180         *       200         *       220         *       240
CcSDR460A1 : AAYFIDFFLHP---HEHRDEVVVYGSGEAKAVLNFEEDVAAYTIKAAIDSRTCDRVIIYRPHENIVSQLQLISLWEKKTG : 233
ObEGS1     : ASYFINYLLRP---YDPKDEITVYGTGEAKFAMNYEQDIGLYTIKVATDPRALNRVVIYRPSTNIITQLELISRWEKKIG : 231
PhEGS1     : AGYFLPTLVQPGATDPPRDKVIISGDGNAKAVFNEEHDIGTYTIKAVDDPRTLNKTLYIKPPKNTLSFNELVAIWEKLIG : 235
CbEGS1     : GAYFVNYLLHPSPHPNRNDDIVIYGTGETKFVLNYEEDIAKYTIKVACDPRCCNRIVIYRPPKNIISQNELISLWEAKSG : 233
CbEGS2     : NGYFLPTLVQPGATTPPRDKVIIPGDGNPKAIFNKEEDIGTYTIKAVDDPRTLNKILYLRPSNNIYSFNELVALWEKKIG : 236
CbIGS1     : GAYFVNYLLHPSPHPNRDDDIVIYGTGETKFVLNYEEDIAKYTIKVACDPRCCNRIVIYRPPKNIISQNELISLWEAKSG : 233
PhIGS1     : TAYFVDYLLHP---RQKSEQVTIYGSGDAKAVLNYEEDVAAYTIKAADDPRAANRVLIIKPPKNIVSQLDLVSSWEKTTG : 234

                        *       260         *       280         *       300         *       320
CcSDR460A1 : QTLKKIHIPEEEVVRLSESLPHPDNIPISILHNIFIKGDQMSFKLKED-DLEATNLYPDYKYTSVDGLLSVCVVD-PPKP : 311
ObEGS1     : KKFKKIHVPEEEIVALTKELPEPENIPIAILHCLFIDGATMSYDFKEN-DVEASTLYPELKFTTIDELLDIFVHD-PPPP : 309
PhEGS1     : KTLEKIYIPEEQILKDIATSPIPINIILAINHSTFVKGDQTNFVIEPSFGVEASELYPDVKYTTVEEYLSHFA------- : 308
CbEGS1     : LSFKKVHMPDEQLVRLSQELPQPQNIPVSILHSIFVKGDLMSYEMRKD-DIEASNLYPELEFTSIDGLLDLFISGRAPPP : 312
CbEGS2     : KTLEKIYVPEEQILKDIQEAPIPINIFLGINHSVFVKGDHTNFEIEPSFGVEASELYPEVKYTTVEEYLDQFV------- : 309
CbIGS1     : LSFKKVHMPDEQLVRLSQELPQPQNIPVSILHSIFVKGDLMSYEMRKD-DIEASNLYPELEFTSIDGLLDLFISGRAPPP : 312
PhIGS1     : STLKMTHISEQEIIKLSESINFPENIHASILHNIFIAGAQLSFELTQDHDLEASELYPNYNYTSVDEYLKICLVN-PPKP : 313

                        *
CcSDR460A1 : KLAAFA---- : 317
ObEGS1     : ASAAF----- : 314
PhEGS1     : ---------- :   -
CbEGS1     : TLAEFE---- : 318
CbEGS2     : ---------- :   -
CbIGS1     : TLAEFE---- : 318
PhIGS1     : KLATYAQPST : 323
```

Supplementary Figure S8. Multiple sequence alignments of EGS and IGS. ObEGS1 (Q15GI4); CbEGS1 (D0VWT0); CbEGS2 (B2WSN0); CbIGS1 (B2WSM8); PhEGS1 (B2WSN1); PhIGS1 (Q15GI3).
